# Contribution of testosterone and estradiol in sexual dimorphism of early-onset Parkinson’s disease

**DOI:** 10.1007/s00702-024-02811-0

**Published:** 2024-07-25

**Authors:** Roberta Bovenzi, Matteo Conti, Clara Simonetta, Jacopo Bissacco, Davide Mascioli, Vito Michienzi, Massimo Pieri, Rocco Cerroni, Claudio Liguori, Mariangela Pierantozzi, Alessandro Stefani, Nicola Biagio Mercuri, Tommaso Schirinzi

**Affiliations:** 1 Department of Systems Medicine, University of Rome “Tor Vergata”, Via Montpellier, Rome, 00133 Italy; 2 Department of Experimental Medicine, University of Rome “Tor Vergata”, Rome, Italy; 3 Department of Clinical Biochemistry, Tor Vergata University Hospital, Rome, Italy; 4 UOSD Parkinson Centre, Tor Vergata University Hospital, Rome, Italy

**Keywords:** Early-onset Parkinson’s disease, Sex, Gender, Sex hormones, Dystonia

## Abstract

Early-onset Parkinson’s disease (EOPD) occurs during the fertile life, when circulating neuroactive sex hormones might enhance the sexual dimorphism of the disease. Here, we aimed to examine how sex hormones can contribute to sex differences in EOPD patients. A cohort of 34 EOPD patients, 20 males and 14 females, underwent comprehensive clinical evaluation of motor and non-motor disturbances. Blood levels of estradiol, total testosterone, follicle-stimulating hormone, and luteinizing hormone were measured in all patients and correlated to clinical features. We found that female patients exhibited greater non-motor symptoms and a relatively higher rate of dystonia than males. In females, lower estradiol levels accounted for higher MDS-UPDRS-II and III scores and more frequent motor complications, while lower testosterone levels were associated with a major occurrence of dystonia. In male patients, no significant correlations emerged. In conclusion, this study highlighted the relevance of sex hormone levels in the sexual dimorphism and unique phenotype of EOPD.

## Introduction

Parkinson’s disease (PD) is a common disorder of the elderly; however, in about 10% of cases, it occurs earlier in life (≤ 50 years of age) as “early-onset PD” (EOPD) (Bovenzi et al. 2023a). EOPD presents specific clinical-pathological traits distinct from those of typical later-onset PD (LOPD), such as slower disease progression or higher incidence of dystonia and levodopa-induced motor complications (Bovenzi et al. 2023a). Moreover, as EOPD affects the prime of life, patients may often complain of marital, social, and occupational issues (Simonetta et al. 2023).

The peculiar sexual dimorphism of PD(Cerri et al. 2019) may be more pronounced in EOPD patients because of the higher circulating levels of sex hormones. Indeed, sex hormones, in particular estradiol and testosterone, have neuroactive properties and probably participate in the pathogenic dynamics of PD (Bovenzi et al. 2023c; Bourque et al. 2024).

Knowledge of sex-related differences in PD mostly comes from studies involving LOPD patients (Vasta et al. 2016; Picillo et al. 2017), while those on EOPD are scarce. Likewise, data regarding the relationship between sex hormone levels and the sex-specific clinical profile of EOPD patients is still lacking.

Therefore, this study aims to examine how sex hormones may contribute to sex differences in EOPD patients, which is critical to understanding the pathophysiological bases of EOPD clinical peculiarities.

## Materials and methods

### Subjects

The study involved 34 EOPD patients, 14 females, and 20 males, all Caucasian, followed up at the Movement Disorders Unit of the Tor Vergata University Hospital (Rome, Italy). Inclusion criteria were diagnosis of PD made by movement disorder specialists following the 2015 MDS Criteria with an age at onset (AAO) > 21 and ≤ 50 years according to the 2022 MDS Task Force recommendations of EOPD (Mehanna 2022). Exclusion criteria were acute or chronic internal disorders, a history of gynecological/prostate malignancy, and use of hormone therapy.

Serum levels of total testosterone (TT), estradiol (E2), and gonadotropins (luteinizing hormone - LH, follicular stimulating hormone - FSH) were measured in all participants, as previously described (Bovenzi et al. 2023c). In females, sex hormone levels were measured during the follicular phase (days 3 to 9) of their menstrual cycle.

At the same time, participants were scored for motor disturbances by the MDS Unified Parkinson’s Disease Rating Scale (MDS-UPDRS) Part II, III, and IV (Goetz et al. 2008), and the Hoehn and Yahr scale (H&Y) (Goetz et al. 2004); for cognition by the mini-mental state examination (MMSE) (Folstein et al. 1975), and the Montreal cognitive assessment (MoCA) (Nasreddine et al. 2005); for non-motor symptoms (NMS) by the Non-motor Symptoms Scale (NMSS) (Chaudhuri et al. 2007); for impulse control disorder (ICD) by the Questionnaire for Impulsive-Compulsive Disorders in Parkinson’s Disease–Rating Scale (QUIP-RS) (Weintraub et al. 2012). Patients were screened for main known PD and parkinsonism-related genes (*SNCA*,* LRRK2*,* VPS35*,* Parkin*,* PINK1*,* PARK7*,* GBA1*,* ATP13A2*,* CHCHD2*,* CP*,* DNAJC6*,* EIF4G1*,* FBOX7*,* MAPT*,* PLA2G6*,* POLG*,* RAB39B*,* SYNJ1*,* SLC6A3*,* VPS13C*) (Cook Shukla et al. 2004) using standard sequencing and multiplex ligation-dependent probe amplification methods. All participants were also grouped into complicated and non-complicated (MDS-UPDRS part IV ≥ 1 vs. 0); dystonic (all forms of dystonia, either as a presenting manifestation of PD or levodopa-induced motor complication) and non-dystonic; ICD-comorbid and non-ICD comorbid (QUIP-RS ≥ 1 vs. 0). The personal levodopa equivalent daily dose (LEDD, mg/day) was calculated for each participant using conventional formula (Schade et al. 2020; Cilia et al. 2023). Clinical assessments were conducted under the effects of habitual medications (“ON” state).

Informed consent was obtained from each participant. The study was performed in agreement with the principles of the Helsinki declarations. The local ethical committee approved the study (protocol number 0026092/2017).

### Statistical analysis

The Kolmogorov-Smirnov test preliminarily assessed normal data distribution. If non-normally distributed, sex hormone levels were Log10-transformed and re-checked for the normal distribution. Qualitative variables between groups were compared using the Chi-square test. Quantitative variables between groups were compared using the one-way-ANCOVA test adjusted for age, AAO, and disease duration. Associations between sex hormone levels and clinical parameters were performed using simple Pearson and partial correlations, using age, disease duration, and AAO as covariates. Logistic regression analysis examined the associations between dichotomic and quantitative variables.

## Results

### Clinical and hormonal sex differences

Table 1 shows the main demographic and clinical features of the population. Females and males were homogeneous in age and disease duration; however, females had lower AAO than males (*p* = 0.04, t=-2.12).

**Table 1:** Demographics, clinical, and biochemical data of the early-onset Parkinson’s disease (EOPD) study population

	Males (*n* = 20)	Females (*n* = 14)	*p*-Value
Age	48.95 ± 5.35	46.29 ± 4.65	NS
Disease duration	3.25 ± 3.97	4.64 ± 3.97	NS
AAO	45.05 ± 5.31	41.29 ± 4.92	*p = 0.042
BMI	25.46 ± 2.08	25.00 ± 2.18	NS
Dystonia (Y/N)	5/15	8/6	NS
ICDs (Y/N) MC (Y/N)	6/14 6/14	7/7 5/9	NS NS
E2	24.7 ± 11.15	129.29 ± 78.95	*p < 0.001
TT	523.35 ± 171.14	26.36 ± 9.74	*p < 0.001
FSH	4.21 ± 2.06	7.30 ± 9.18	NS
LH	2.59 ± 1.43	4.68 ± 6.30	NS
H&Y	2.10 ± 0.42	2.18 ± 0.75	NS
MDS UPDRS II	9.13 ± 7.10	11.20 ± 9.37	NS
MDS UPDRS III	28.421 ± 9.00	29.21 ± 11.80	NS
MDS UPDRS IV	1.47 ± 2.39	3.86 ± 6.26	NS
NMSS	21.61 ± 21.16	36.86 ± 19.96	*p = 0.046
NMSS 1	0.67 ± 1.65	2.08 ± 2.63	NS
NMSS 2	3.22 ± 3.98	8.77 ± 6.08	*p = 0.005
NMSS 3	5.28 ± 5.41	5.31 ± 7.00	NS
NMSS 4	0.00 ± 0.00	0.15 ± 0.55	NS
NMSS 5	0.89 ± 2.11	0.53 ± 1.198	NS
NMSS 6	1.72 ± 2.55	4.77 ± 3.83	*p = 0.013
NMSS 7	4.20 ± 5.73	4.00 ± 4.93	NS
NMSS 8	1.00 ± 2.47	4.00 ± 4.67	*p = 0.050
NMSS 9 MMSE MoCA	2.72 ± 4.06 29.44 ± 0.96 26.75 ± 2.57	5.46 ± 5.95 29.70 ± 0.67 26.33 ± 3.52	NS NS NS
QUIP-RS LEDD	2.10 ± 4.48 387.05 ± 406.33	2.92 ± 5.73 410.21 ± 421.22	NS NS

n, number; AAO, age at onset; BMI, Body Mass Index (kg/m^2^); MC, motor complications; H&Y, Hoehn & Yahr Scale; MDS-UPDRS, Movement Disorder Society Unified Parkinson’s Disease Rating Scale; NMSS, Non Motor Symptoms Scale; MMSE, Mini Mental State Examination; MoCA, Montreal Cognitive Assessment Scale; QUIP-RS, Questionnaire for Impulsive-Compulsive Disorders in Parkinson’s Disease–Rating Scale. Age, age at onset, and disease duration are expressed in years. Total testosterone is expressed in ng/dL, Estradiol in pg/ml, FSH in mIU/mL and LH in mIU/mL. *Significant differences between groups; NS: non significative test

Table 2 shows the main genetic findings of the EOPD cohort; no differences were found in the rate of monogenic forms between the two sexes.

**Table 2:** The table shows the main genetic findings of the EOPD cohort

	Males (*n* = 20)	Females (*n* = 14)
*LRRK2*	1 heterozygous G2019S	-
	1 heterozygous R1441C	-
*GBA1*	-	1 heterozygous L444P
*Parkin*	1 homozygous c.1244 C > A; (p.8Thr415Asn)	-
	1 heterozygous c.245 C > A; p.(Ala82Glu) (VUS)	1 heterozygous c.719 C > T; 1 p.(Thr240Met)
		1 heterzozygous exon 3 deletion
*PARK7*	-	1 homozygous c.200 A > G;p(Tyr67Cys) (VUS)
*SYNJ1*	1 heterozygous c.1915 A > G; p.(lle639Val) (VUS)	-
*POLG*	1 heterozygous c.241 A > G p.(Arg81Gly) het (VUS)	-
No mutations	14	10

*LRRK2*, leucine-rich repeat kinase 2; *GBA*1, glucosidase beta acid 1; *PARK7*, parkinsonism associated deglycase; *SYNJ1;* synaptojanin-1; *POLG*, DNA polymerase gamma

Female EOPD patients had higher NMSS total scores than males (*p* = 0.046, t=-2.08), even in the adjusted model (*p* = 0.026, F = 5.57). Significant differences resulted in the “sleep/fatigue” (*p* = 0.027, F = 5.46), the “gastrointestinal tract” (*p* = 0.019, F = 6.23), and the “sexual function” domains (*p* = 0.05, F = 4.08).

No intersex differences emerged in motor scores, prevalence of motor complications, and LEDD. Dystonia was more prevalent in females, near to statistical significance (Chi-square statistics 3.6028, *p* = 0.058). ICD comorbidity and severity were similar between the two sexes.

Female EOPD patients had higher E2 levels and lower TT levels than males (*p* < 0.001, t=-8.76 and *p* < 0.001, t = 22.45, respectively). Gonadotropin levels were higher in females than males, although not significantly.

### Correlation analysis in female EOPD patients

None of the sex hormone levels correlated with the NMSS total score. FSH levels positively correlated with the “gastrointestinal tract” domain (*r* = 0.628, *p* = 0.021), although the significance was lost in the adjusted model.

**Fig. 1:**
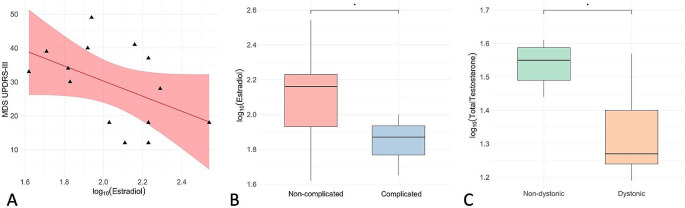
The figure shows the main findings within the female early-onset Parkinson’s disease (EOPD) study population. **Plot A** shows the significant inverse correlation between estradiol levels and MDS-UPDRS-III scores. **Plot B** shows the significant difference in estradiol levels between complicated and non-complicated patients. **Plot C** shows the significant difference in total testosterone levels between dystonic and non-dystonic patients. Estradiol (pg/ml) and total testosterone (ng/dL) levels are log_10_-transformed

E2 levels negatively correlated with MDS-UPDRS part II and III total scores (*r*=-0.772, *p* = 0.042; *r* = 0.227, *p* = 0.010, respectively) (Fig. 1, **Plot A**). Moreover, E2 levels were significantly lower in complicated female patients than in non-complicated ones (78.0 ± 11.53 vs. 143.27 ± 84.09, F = 2.268, *p* = 0.03) (Fig. 1, **Plot B**).

TT levels inversely correlated with MDS-UPDRS part IV scores (*r*=-0.621, *p* = 0.05) in the adjusted model but not in the simple one. Female patients with dystonia had lower TT levels than patients without (21.51 ± 8.40 vs. 32.0 ± 8.46, F = 0.232, *p* = 0.043) (Fig. 1, **Plot C**). The logistic regression analysis using each sex hormone (TT, E2, LH, and FSH) to predict the occurrence of dystonia adjusting for age, AAO, and LEDD, demonstrated that only TT was significantly associated (r^2^ = 0.65, beta=-15.170, *p* = 0.022). The same analysis was performed to predict the occurrence of motor complications and ICD, with no significant results.

E2 levels positively correlated with MoCA scores (*r* = 0.619, *p* = 0.032), although the significance was lost in the adjusted model.

No correlations were found between sex hormones and BMI.

### Correlation analysis in male EOPD patients

Sex hormones did not correlate with any variable and did not differ between clinical groups (dystonia, motor complications, ICD). The logistic regression analysis using each sex hormone level to predict the occurrence of dystonia, motor complications, or ICD, adjusting for age, AAO, and LEDD, did not provide significant results.

## Discussion

This study investigating EOPD sex differences and their relations with sex hormone levels found distinct clinical-hormonal profiles in female and male patients, suggestive of sex-specific pathophysiological mechanisms.

Indeed, albeit within a small and homogeneous cohort, we showed that female EOPD patients suffered from a greater NMS burden and a relatively higher rate of dystonia compared to males and that, in females, estradiol and testosterone were significantly associated with these features.

The finding of a higher prevalence of NMS in female EOPD patients basically disagrees with previous literature on typical LOPD (Russillo et al. 2022), although the prominence of sleep(Russillo et al. 2022; Bovenzi et al. 2024a) and gastrointestinal disturbances(Cerri et al. 2019) in females have already been observed. Also, the sexual issues resulted as unexpected. In fact, dissatisfaction with sexual life, as well as cognitive decline, are major complaints in male patients (Russillo et al. 2022). Such impairment in sexual function among EOPD females might represent a peculiarity of the EOPD condition, arising from psychosocial and medical factors (such as the absence of supporting drugs) rather than from the hormonal status. Cognition, instead, was equally preserved between the two sexes, in line with the young age and the short disease duration of the cohort (Bovenzi et al. 2023a; Simonetta et al. 2023).

Sex hormone levels were unrelated to the NMS sphere, either in females or males. Although some raw association resulted in females between estradiol and cognition and FSH and gastrointestinal dysfunction, the adjusted analyses excluded significant relations.

Conversely, sex hormones significantly contributed to the dimorphism in motor disturbances. Here, the severity of the motor syndrome was mild and similar between sexes, probably due to the relatively short disease duration. However, the prevalence of dystonia, either as a PD-presenting or a motor fluctuation sign, was higher in females, albeit not reaching the significance threshold.

Dystonia is a common manifestation of EOPD, especially in female patients (Jankovic and Tintner 2001), although in the absence of clear biological bases. Here, we noticed that testosterone might play a role. We measured lower testosterone circulating content in female EOPD patients with dystonia compared to those without and observed that testosterone levels predicted either dystonia occurrence or the severity of motor complications (as MDS-UPDRS IV score). Of relevance, no significant associations resulted among males.

This data, albeit new, is supported by experimental evidence. Indeed, in a dystonia animal model, the dystonic attacks were reduced by the puberty-related increase in gonadal testosterone production (Löscher et al. 1995). Testosterone deficiency affects cerebellar networks in humans, impairing the vestibular-ocular reflex (VOR) (Panichi et al. 2022), which is known to be compromised in dystonic patients (Stell et al. 1989), while hypogonadal male patients with Klinefelter syndrome may suffer from dystonic tremor, partially improved by exogenous testosterone replacement (Rabin et al. 2015). Finally, sex hormones and testosterone (Löscher et al. 1995) might modulate central GABAergic transmission (Butler et al. 2015; Conte et al. 2017), which is critical in the pathophysiology of dystonia (Schirinzi et al. 2018).

Therefore, a given threshold of circulating testosterone seems to be critical for motor brain circuits. Accordingly, in EOPD females, whose testosterone levels are physiologically lower than males, a further reduction may trigger dystonia (or dyskinesia) onset, justifying a possible greater predisposition to such phenomena in women.

Estradiol levels were inversely associated with other motor disturbances in females. Female EOPD patients with lower estradiol levels indeed had more severe motor impairment (as MDS-UPDRS part II and III scores) and major occurrence of motor complications. This is in line with the pro-dopaminergic and neuroprotective effects of estradiol (Cerri et al. 2019), largely demonstrated in preclinical settings and in female (even postmenopausal) (Bovenzi et al. 2023a, b, 2024b) and male (Bovenzi et al. 2023c) PD patients.

ICD is a main issue in EOPD and is commonly more prevalent in males (Cerri et al. 2019). Accordingly, we would have expected some sex differences or hormone-dependent mechanisms in our cohort. Conversely, both the number of ICD-comorbid patients and the ICD severity (as QUIP-RS score) were similar between the two sexes, possibly because of the short disease duration. As well, no significant associations resulted with sex hormones.

Undoubtedly, genetics play an important role in EOPD, and sex might influence the penetrance of certain forms of genetic PD. Some studies have reported a higher prevalence of *LRRK2* and *GBA* pathogenic variants in female PD patients (Cilia et al. 2014; Alcalay et al. 2018; Li et al. 2021). In our cohort, monogenic cases were relatively few and equally distributed between the two sexes, thus preventing speculations on a putative role for genetic clustering in the sexual dimorphism observed.

This study has several limitations mainly due to the small sample size, the cross-sectional design, the population features (enrollment of patients with short disease duration, poor clinical assessment for levodopa-induced complications, and non-motor fluctuations (Donzuso et al. 2023), the lack of comparative LOPD and healthy controls groups.

Nevertheless, we highlighted some sex hormone-based differences in EOPD. Larger studies, eventually prospective, including neurophysiological assessments, and control groups, are now due to confirm and extend these still preliminary findings.

## Data Availability

The datasets generated during analysis are available from the corresponding author upon reasonable request.
